# Volatile Organic Compounds (VOCs) Produced by *Gluconobacter cerinus* and *Hanseniaspora osmophila* Displaying Control Effect against Table Grape-Rot Pathogens

**DOI:** 10.3390/antibiotics10060663

**Published:** 2021-06-01

**Authors:** Ninoska Delgado, Matías Olivera, Fabiola Cádiz, Guillermo Bravo, Iván Montenegro, Alejandro Madrid, Claudia Fuentealba, Romina Pedreschi, Eduardo Salgado, Ximena Besoain

**Affiliations:** 1 Escuela de Agronomía, Facultad de Ciencias Agronómicas y de los Alimentos, Pontificia Universidad Católica de Valparaíso, San Francisco s/n La Palma, Quillota 2260000, Chile; matias.olivera.a@gmail.com (M.O.); fabiola.cadiz@pucv.cl (F.C.); romina.pedreschi@pucv.cl (R.P.); eduardo.salgado@pucv.cl (E.S.); 2 Molecular Microbiology and Environmental Biotechnology Laboratory, Department of Chemistry & Center of Biotechnology Daniel Alkalay Lowitt, Universidad Técnica Federico Santa María, Avda. España 1680, Valparaíso 2390123, Chile; bravoc.guillermo@gmail.com; 3 Escuela de Obstetricia y Puericultura, Facultad de Medicina, Universidad de Valparaíso, Angamos 655, Reñaca, Viña del Mar 2520000, Chile; ivan.montenegro@uv.cl; 4 Laboratorio de Productos Naturales y Síntesis Orgánica (LPNSO), Departamento de Química, Facultad de Ciencias Naturales y Exactas, Universidad de Playa Ancha, Avda. Leopoldo Carvallo 270, Playa Ancha, Valparaíso 2340000, Chile; alejandro.madrid@upla.cl; 5 Escuela de Alimentos, Facultad de Ciencias Agronómicas y de los Alimentos, Pontificia Universidad Católica de Valparaíso, Waddington 716, Valparaíso 2340000, Chile; claudia.fuentealba@pucv.cl

**Keywords:** bioantagonist, volatile organic compounds, table grapes, mycelial inhibition, biocontrol, summer bunch rot

## Abstract

Table grapes (*Vitis vinifera*) are affected by botrytis bunch rot and summer bunch rot, the latter a complex disease caused by *Botrytis cinerea*, *Aspergillus* spp., *Penicillium expansum* and *Rhizopus stolonifer*. To search for biocontrol alternatives, a new bioproduct composed of *Gluconobacter cerinus* and *Hanseniaspora osmophila*, a consortium called PUCV-VBL, was developed for the control of fungal rots in table grapes. Since this consortium presents new biocontrol species, the effect of their VOCs (volatile organic compounds) was evaluated under in vitro and in vivo conditions. The VOCs produced by the PUCV-VBL consortium showed the highest mycelial inhibition against *Botrytis cinerea* (86%). Furthermore, *H. osmophila* was able to inhibit sporulation of *A. tubingensis* and *P. expansum*. VOCs’ effect in vivo was evaluated using berries from Red Globe, Thompson Seedless and Crimson Seedless grapes cultivars, demonstrating a mycelial inhibition by VOCs greater than 70% for all evaluated fungal species. The VOC identification of the PUCV-VBL consortium was analyzed by solid-phase microextraction coupled to gas chromatography-mass spectrometry (SPME-GCMS). A total 26 compounds were identified, including 1-butanol 3-methyl, propanoic acid ethyl ester, ethyl acetate, phenylethyl alcohol, isobutyl acetate and hexanoic acid ethyl ester. Our results show that VOCs are an important mode of action of the PUCV-VBL biological consortium.

## 1. Introduction

Summer bunch rot complex and botrytis bunch rot (“gray mold”) are responsible for the most significant reductions in table grape yield and quality worldwide [1]. In late summer, these microorganisms along with bacteria and yeasts, cause sour rot, a polymicrobial disease that attacks ripe, thin-skinned grapes [2]. In Chile, *Botrytis cinerea* pathologies have been shown to seriously affect susceptible cultivars, such as Thompson Seedless [3]. Besides, it was determined that the causal agents associated with summer bunch rot in cv. Red Globe is mainly due to *Botrytis cinerea*, *Aspergillus* spp., *Rhizopus stolonifer* and *Penicillium expansum* [4]. These pathogens are distributed worldwide, and infection can occur during the growing season, harvest, postharvest, storage, or transport [5,6].

Especially in postharvest, these diseases can cause significant economic losses: it is estimated that, in developed countries, 25% of fruit can be affected by such pathogens; and that, in developing countries, the percentage doubles [7], accounting for 80% of postharvest losses [8]. Chile, which is the most important table grape exporting country globally [9], these diseases can cause severe difficulties, especially when reaching the Far East destination market.

In this context, fungicides are the most common control method for these pathogens [10]; indeed, table grape and wine sectors represent 50% of the total market value for fungicides [10]. However, fungicide resistance is a growing problem [11,12,13,14,15]. Botrytis management’s overall expenses, including cultural measures, botrycides, broad-spectrum fungicides, and biocontrols, easily reach €1 billion/year in all countries; product and quality loss in the market chain, however, is probably much higher [10].

While Regulation (EU) 2015/408 [16] has proposed a chemical substitution list currently used in traditional agriculture, these alternative methods still require development and evaluation to demonstrate at least the same effectiveness in controlling postharvest diseases. With this goal, physical, chemical, and biological (biocontrol) control methods have been developed as alternatives to pesticides [14].

Of the above, biocontrol methods seek to decrease microbial pathogen inoculum or remit disease through one or more yeast, bacterial, and or fungal microorganisms [17]. Over the past 30 years, biocontrol research by multidisciplinary scientific teams, agri-food companies in the agri-sector and multinationals chemical companies [18] has produced phytosanitary compounds and contributed to its becoming an effective strategy to combat postharvest decay of fruits. Research into, e.g., yeast biocontrol methods have shown promising results, demonstrating ideal antagonistic properties and adaptability to adverse environmental conditions, few nutritional requirements, and prolonged half-life formulation [19,20]. Although numerous antifungal biocontrol procedures have been developed and patented in several countries [18,21,22,23], very few have been applied to agricultural use. This is likely due to their low antagonistic effectiveness not meeting the high quality and safety requirements, and trade regulations, of international markets [18].

In addressing the above, a promising area of biocontrol research in postharvest disease control is that of volatile organic compounds (VOCs), which have been shown to play a significant role in the control of several fungal pathogens [24,25,26]. Produced by microorganisms at very low concentrations [27], VOCs—which are biodegradable and do not leave toxic residues on fruit surfaces [28,29]—are hydrophobic, organic molecules with a low molecular weight (<300 Da) and high vapor pressure (≥0.01 kPa at 20 °C) [30]. The majority of VOCs, which can be created (refining, evaporation of organic solvents, unburned, etc.) or are naturally occurring (emissions by plants, animals, and microorganisms), belong to five chemical groups: terpenoids, fatty acid derivatives, benzenoid compounds, phenylpropanoids, and amino acid derivatives. Furthermore, VOCs produced naturally diffuse through biological fumigation (or “biofumigation”), which has shown promise against a wide range of storage pathogens and fungal decay [16,22].

To continue advances in the field of “clean technology” fungicide alternatives, the PUCV-VBL biological consortium research group developed a bioproduct with the ability to control table grape diseases, formulated with two antagonistic microorganisms isolated from table grapes bunches, *Gluconobacter cerinus* strain 515 and *Hanseniaspora osmophila* strain 337, for the control of fungi in grapes (WO2017088081A1).

Therefore, it is postulated that *G. cerinus* and *H. osmophila* possess VOCs as a mode of action capable of inhibiting the mycelial growth of pathogens that cause rot in table grapes. The objectives of this work were to evaluate the effect of the VOCs produced by the PUCV-VBL consortium; specifically, the following were studied: (i) the in vitro effects on *Aspergillus*, *Botrytis*, *Penicillium* and *Rhizopus* fungi; (ii) the in vivo effects against pathogens in Thompson Seedless, Crimson Seedless, and Red Globe cultivars; and (iii) the VOCs shown to control pathogenic fungi in table grapes in previous stages were characterized through Solid Phase Microextraction followed by Gas Chromatography-Mass Spectrometry (SPME-GC-MS).

## 2. Results

### 2.1. In Vitro Assay of VOC Production

**Figure 1:**
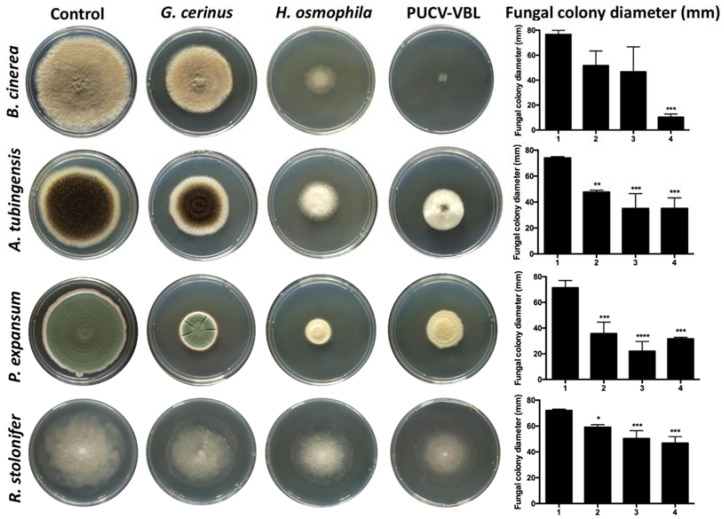
Effect of VOCs of bioantagonists *G. cerinus*, *H. osmophila* and PUCV-VBL biological consortium on mycelial growth of pathogen *B. cinerea*, *A. tubingensis*, *P. expansum* and *R. stolonifer* under in vitro conditions, established by means of a double plate assay and through fungal colony diameter graph (mm) measurement, conducted using image analysis with the Image J^®^ progam. 1: Control without biocontroller, 2: VOCs of *G. cerinus*, 3: VOCs of *H. osmophila*, 4: VOCs of PUCV-VBL biological consortium. Tukey’s multiple comparisons test * *p* < 0.05; ** *p* < 0.01; *** *p* < 0.001; **** *p* < 0.0001.

**Scheme 1:**
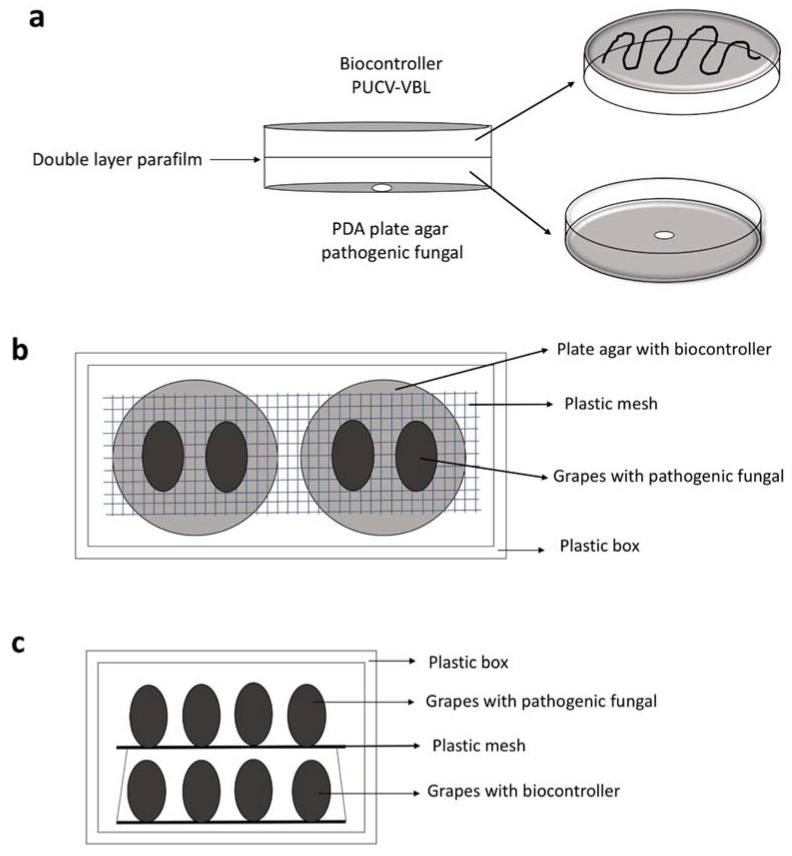
Schematic representation in vitro and in vivo tests. (**a**) Double plate test. (**b**) In vivo test berries inoculated with pathogens on Petri dishes with biocontroller. (**c**) In vivo test berries of grapes inoculated with pathogens on grape berries inoculated with biocontroller.

In Figure 1, the in vitro effects of VOCs from bioantagonists *G. cerinus* and *H. osmophila* on the pathogens *Botrytis cinerea*, *Aspergillus tubingensis*, *Penicillium expansum* and *Rhizopus stolonifer*, as determined by double plate test (Scheme 1a), are shown. The VOCs emitted by the bioantagonists were shown to effectively inhibit mycelial growth of all pathogens evaluated. Indeed, inhibitory effects were greater in the PUCV-VBL biocontrol than those of each BCAs separately, demonstrating a synergistic effect among all the VOCs.

*Botrytis cinerea* was most affected, at 86% inhibition mycelial growth (Table 1). *Aspergillus tubingensis* was significantly inhibited as well, achieving 52%. This is likely due to *H. osmophila* VOCs preferentially inhibiting *A. tubingensis* sporulation instead of mycelial growth. *Penicillium expansum* also showed significant inhibition (68%), and similar to *A. tubingensis*, *H. osmophila* VOCs inhibited sporulation of *P. expansum*. While mycelia of *Rhizopus stolonifer* was the least inhibited (35%), it still had significant differences concerning control.

### 2.2. In Vivo Assays of VOC

**Figure 2:**
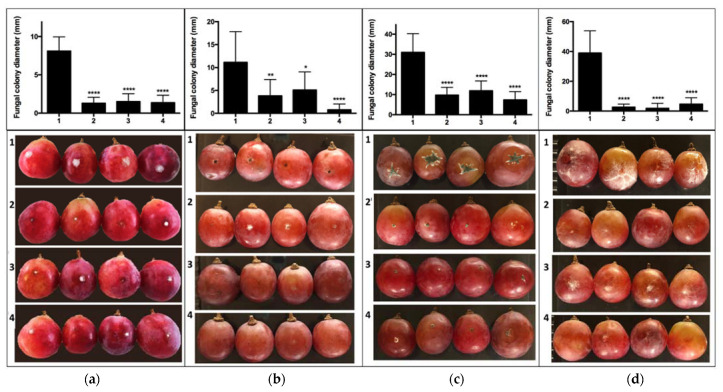

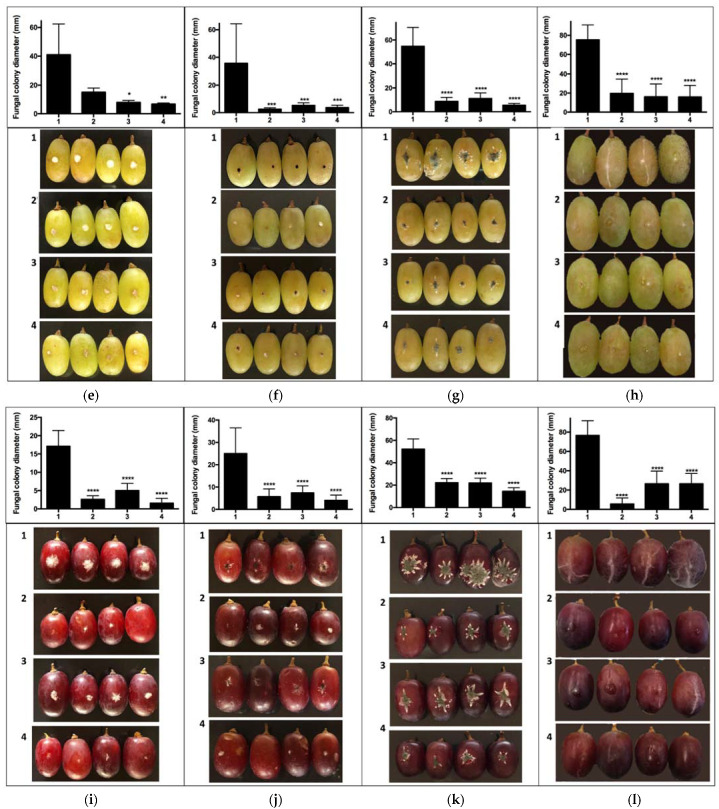
In vivo test berries inoculated with pathogens on Petri dishes with biocontroller. Effect of VOCs produced by (1) control treatment without biocontroller; (2) *H. osmophila* biocontroller; (3) *G. cerinus* biocontroller; (4) Biological consortium PUCV-VBL biocontroller. (**a**) Percentage of mycelial inhibition of *B. cinerea* in cv Red Globe, (**b**) percentage of mycelial inhibition of *A. tubingensis* in cv Red Globe, (**c**) percentage of mycelial inhibition of *P. expansum* in cv Red Globe, (**d**) percentage of mycelial inhibition of *R. stolonifer* in cv Red Globe, (**e**) percentage of mycelial inhibition of *B. cinerea* in cv Thompson Seedless, (**f**) percentage of mycelial inhibition of *A. tubingensis* in cv Thompson Seedless, (**g**) percentage of mycelial inhibition of *P. expansum* in cv Thompson Seedless, (**h**) percentage of mycelial inhibition of *R. stolonifer* in cv Thompson Seedless, (**i**) percentage of mycelial inhibition of *B. cinerea* in cv Crimson Seedless, (**j**) percentage of mycelial inhibition of *A. tubingensis* in cv Crimson Seedless, (**k**) percentage of mycelial inhibition of *P. expansum* in cv Crimson Seedless, (**l**) percentage of mycelial inhibition of *R. stolonifer* in cv Crimson Seedless. Tukey’s multiple comparisons test * *p* < 0.05; ** *p* < 0.01; *** *p* < 0.001; **** *p* < 0.0001.

Of the two in vivo trials conducted for VOC production, the results of the first BCAs in a Petri dish underneath grapes inoculated with pathogens (Scheme 1b) showed significant differences in mycelial growth of all pathogens, for the three grape cultivars evaluated, compared to control (Figure 2). For cv. Red Globe, the most significant effect was against *A. tubingensis*, at 93% mycelial inhibition (Table 2). There, *G. cerinus* was shown to inhibit its sporulation as well. Similar results were observed for cv. Thompson Seedless, at 79% mycelial inhibition for *B. cinerea* (statistically significant, Table 2). For cv. Crimson Seedless, the PUCV-VBL biocontrol VOCs presented the same level of significance against *A. tubingensis*, *P. expansum* and *R. stolonifer*. Only for *R. stolonifer* was the effect slightly less (Table 2).

**Figure 3:**
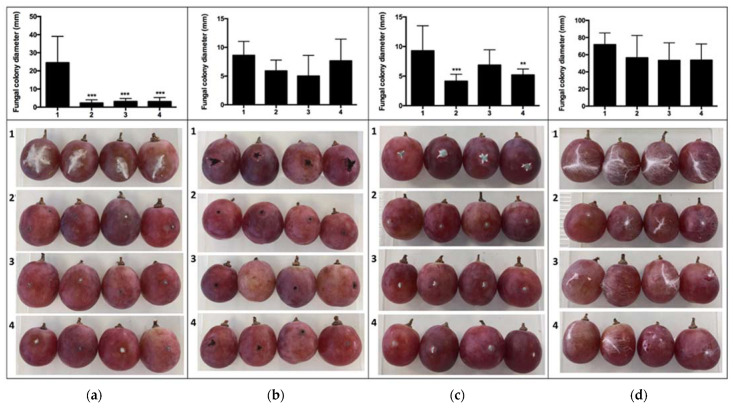

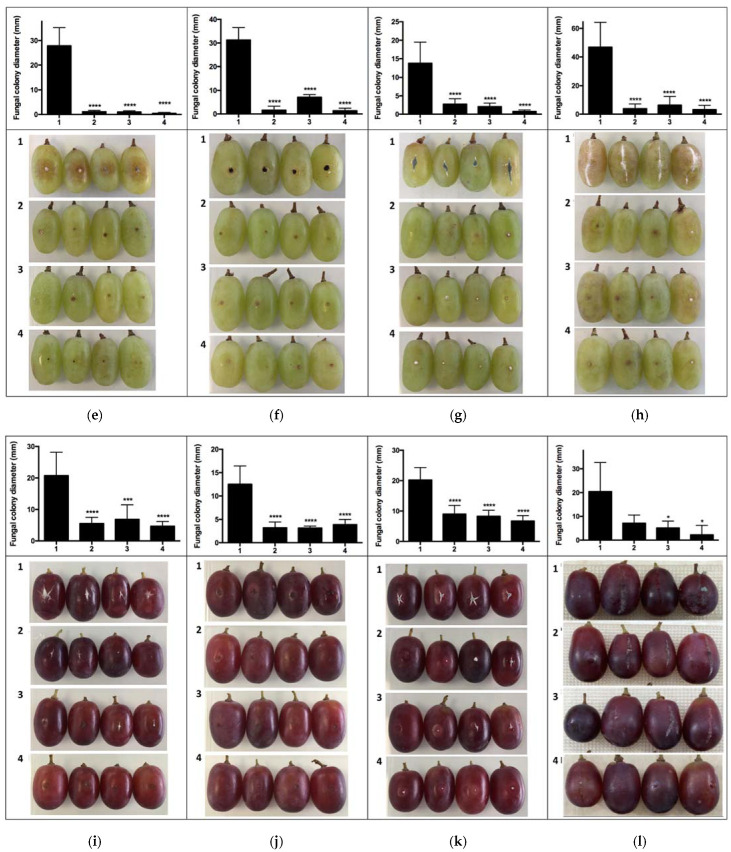
In vivo test berries of grapes inoculated with pathogens on grape berries inoculated with biocontroller. Effect of VOCs produced by (1) control treatment without biocontroller; (2) *H. osmophila* biocontroller; (3) *G. cerinus* biocontroller; (4) Biological consortium PUCV-VBL biocontroller. (**a**) Percentage of mycelial inhibition of *B. cinerea* in cv Red Globe, (**b**) percentage of mycelial inhibition of *A. tubingensis* in cv Red Globe, (**c**) percentage of mycelial inhibition of *P. expansum* in cv Red Globe, (**d**) percentage of mycelial inhibition of *R. stolonifer* in cv Red Globe, (**e**) percentage of mycelial inhibition of *B. cinerea* in cv Thompson Seedless, (**f**) percentage of mycelial inhibition of *A. tubingensis* in cv Thompson Seedless, (**g**) percentage of mycelial inhibition of *P. expansum* in cv Thompson Seedless, (**h**) percentage of mycelial inhibition of *R. stolonifer* in cv Thompson Seedless, (**i**) percentage of mycelial inhibition of *B. cinerea* in cv Crimson Seedless, (**j**) percentage of mycelial inhibition of *A. tubingensis* in cv Crimson Seedless, (**k**) percentage of mycelial inhibition of *P. expansum* in cv Crimson Seedless, (**l**) percentage of mycelial inhibition of *R. stolonifer* in cv Crimson Seedless. Tukey’s multiple comparisons test * *p* < 0.05; ** *p* < 0.01; *** *p* < 0.001; **** *p* < 0.0001.

The second in vivo trial results—BCAs in grapes placed underneath pathogen-inoculated berries (Scheme 1c)—showed significant differences for *B. cinerea* and *P. expansum* in cv. Red Globe (Figure 3a,c); and, for cvs. Thompson Seedless and Crimson Seedless, all treatments differed from control for all the pathogens evaluated (Figure 3e–l).

No statistical differences were observed for cv. Red Globe among the three separate treatments (Table 3); however, for cv. Thompson Seedless, treatment PUCV-VBL biocontrol VOCs was significantly more effective against *A. tubingensis* and *P. expansum* (over 90% inhibition) (Table 3). For cv. Crimson Seedless, the most considerable effect was for PUCV-VBL biocontroller VOCs, at 72% mycelial inhibition for *B. cinerea* (statistically significant, Table 3).

### 2.3. Identification of VOCs Produced by BCAs (SPME GC-MS)

**Figure 4:**
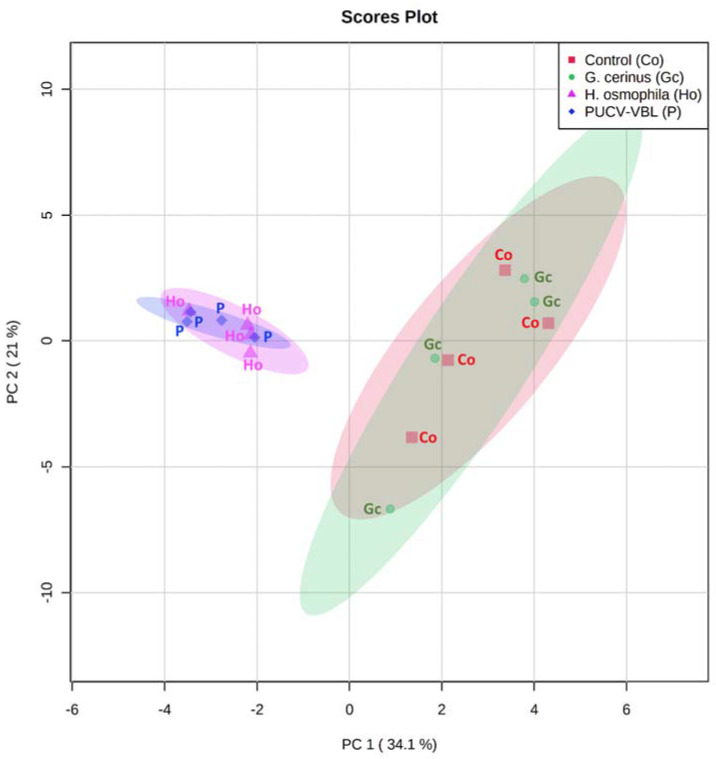
Principal Component Analysis (PCA) of SPME-GCMS data. PCA score plot. Control (Co), grape without biocontroller, red; G. cerinus (Gc), grape with biocontroller *G. cerinus*, green; H. osmophila (Ho), grape with biocontroller *H. osmophila*, magenta; PUCV-VBL (P), grape with PUCV-VBL consortium, violet.

**Figure 5:**
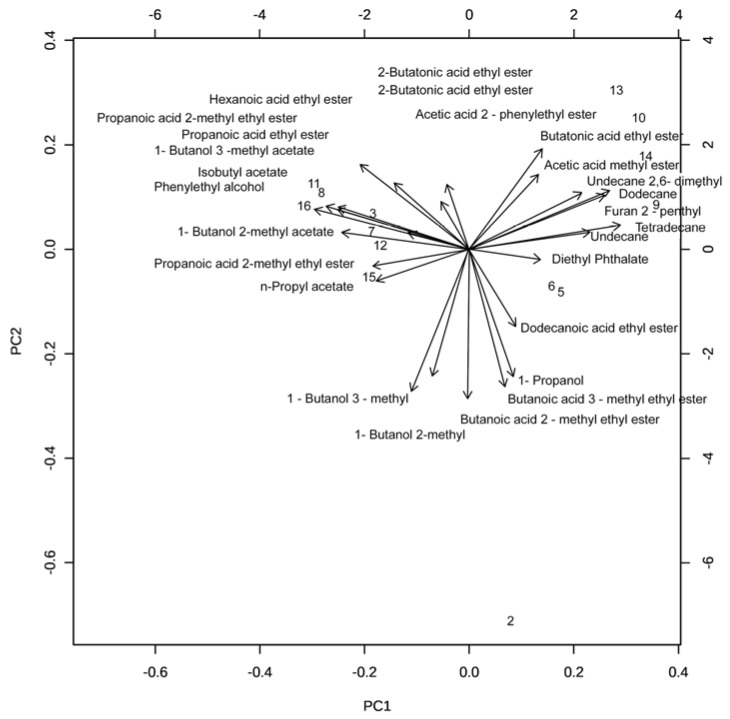
Principal Component Analysis (PCA) Biplot of SPME-GCMS data, volatile organic compounds (VOCs) of biological consortium PUCV-VBL.

**Figure 6:**
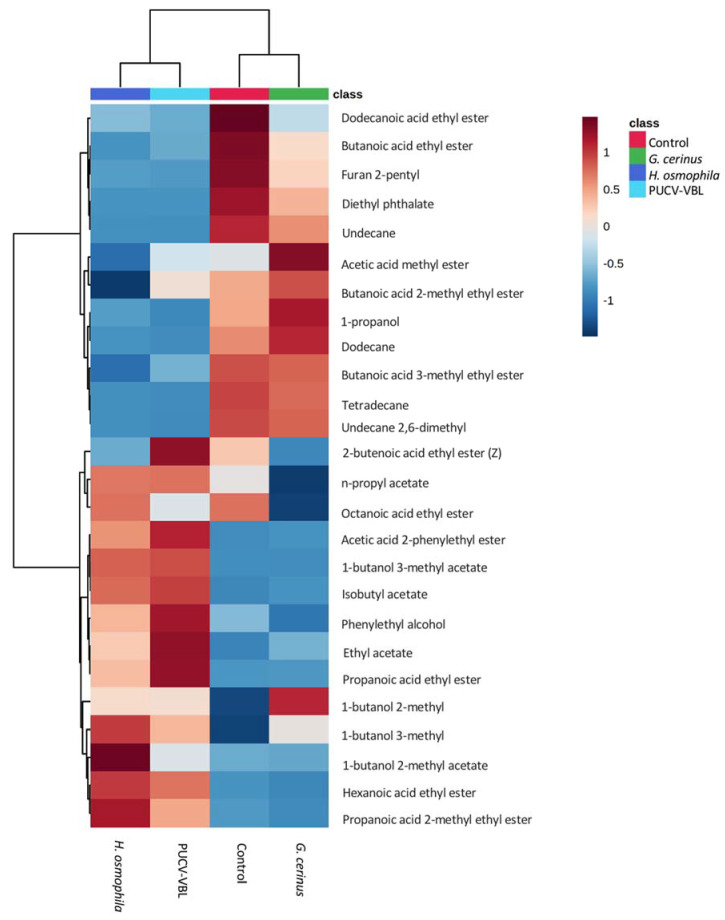
Heat map based on Euclidean distance using Ward algorithm and showing group averages.. Each row in the color heat map indicates a single compound. Blue to reddish in the color code indicates down-to-up relative concentration of the compounds. Control, grape without biocontroller, red; *G. cerinus* grape with biocontroller *G. cerinus*, green; *H. osmophila*, grape with biocontroller *H. osmophila*, blue; PUCV-VBL, grape with PUCV-VBL consortium, light blue.

A total of 26 VOCs produced by bioantagonists *G. cerinus*, *H. osmophila*, and the combined PUCV-VBL biocontrol taken directly from grapes were identified in different concentrations by SPME GC-MS. Figure 4 shows the Principal Component Analysis (PCA) projection for biocontroller distributions regarding PC-1 with PC-2. BCAs metabolites are shown in four groups. *H. osmophila* metabolites are most represented in the PUCV-VBL biocontrol. Figure 5 shows a PCA biplot of VOCs correlated with the four treatments. Figure 6 shows a Euclidean distance heat map (Ward algorithm) of replicas for each treatment for the presence of all VOC compounds produced by bioantagonists, where dark brown indicates higher relative concentrations and dark blue, the lowest.

Results show that *Hanseniaspora osmophila* is most responsible for VOCs in grapes (1-butanol 3-methyl, 2-methyl butanoic acid, 2-methyl 1-butanol, hexanoic ethyl acid, 3-methyl 1-butanol, 2-phenyl acetic acid, propyl acetate, phenylethyl alcohol, ethyl acetate, 2-propanoic acid, isobutyl acetate, and propanoic acid); however, the same VOCs were present in the PUCV-VBL biocontrol. Although *G. cerinus* had fewer overall contributions to VOCs, it did overexpress 2-butenoic acid (also present in the PUCV-VBL biocontrol) and methyl acetic acid. There was some overlap in *G. cerinus* and control VOCs: 2-methyl-1-butanol, undecane, 1-propanol, dodecane, 2,6-dimethyl undecane, butanoic ethyl acid, 3-methyl butanoic acid, and tetradecane.

**Figure 7:**
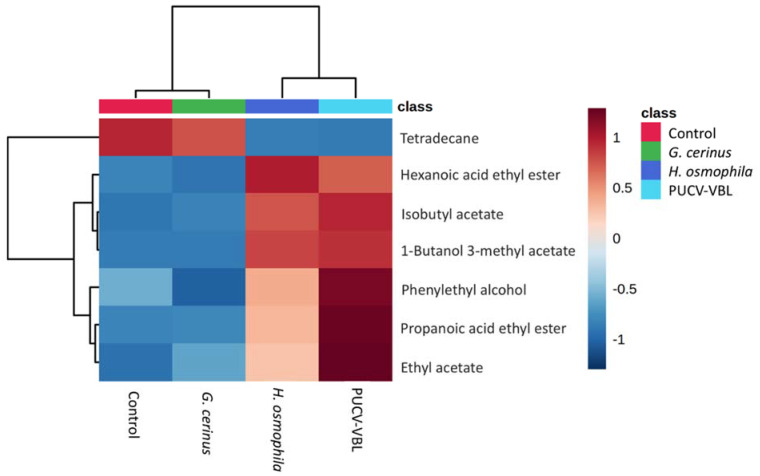
Heat map with significant VOCs ANOVA (FDR = 0.05) FDR = False Discovery Rate. Control, grape without biocontroller, red; *G. cerinus*, grape with biocontroller *G. cerinus*, green; *H. osmophila*, grape with biocontroller *H. osmophila* blue; PUCV-VBL, grape with PUCV-VBL consortium, light blue. Blue to reddish in the color code indicates down- to up-regulation of the compounds.

Finally, the VOCs significantly more present in both *H. osmophila* and the PUCV-VBL biocontrol were 1-butanol 3-methyl, propanoic acid ethyl ester, ethyl acetate, phenylethyl alcohol, isobutyl acetate, and hexanoic acid ethyl ester. Tetradecane was present only in control and *G. cerinus* treatments (Figure 7).

## 3. Discussion

The PUCV-VBL biocontrol was shown to produce a wide range of VOCs. Desirable for their volatile characteristics and diffusibility in the air [31,32], VOCs are typically smaller than other BCAs secondary metabolites (up to C20) with low molecular mass (100-500 Daltons), high vapor pressure, low boiling point, and lipophilia [33]. These properties facilitate evaporation and diffusion through water- and gas-filled pores in soil and rhizosphere environments [34,35].

The present work shows that PUCV-VBL biocontrol VOCs limited the mycelial growth of fungal pathogens in table grapes. For in vitro, *B. cinerea* was most inhibited, at 86%. Next, VOCs emitted by component BCA *H. osmophila* were shown to inhibit sporulation of *A. tubingensis* and *P. expansum* (Figure 1). This is similar to the results obtained by Ul Hassan et al., [36] for *Bacillus licheniformis* VOCs, who indicated that, among them, 3-methyl-1-butanol was most responsible for inhibiting mycelial growth and sporulation. Our results also showed 3-methyl-1-butanol as the predominant VOC molecule produced by *H. osmophila*. Interestingly, some *Bacillus* spp.—including *B. subtilis*, *B. amyloliquefaciens*, and *B. cereus*—have been reported to produce 3-methyl-1-butanol and act as a robust antifungal compound [37].

SPME-GC-MS was used to identify VOCs from the PUCV-VBL biocontrol and its BCAs components, *G. cerinus* and *H. osmophila*. VOCs were detected after 72 h of bioantagonist incubation. Principal component analysis (PCA) of SPME-GC-MS VOC data revealed heterogeneity in component contribution to VOCs (Figure 4 and Figure 5). VOCs were similar between control and *G. cerinus* and between *H. osmophila* and the PUCV-VBL biocontrol. A Euclidean distance heat map based on the Ward algorithm for each treatment’s replicas provides an intuitive visualization of all compounds (Figure 6). Both component BCAs produced the same compounds but with different relative peak areas, and they are present in different concentrations. There are two distinguishable VOC clusters: that of the PUCV-VBL biocontrol and *H. osmophila*, in which VOC are overexpressed; and that of *G. cerinus* and the control, with lesser concentrations.

The VOCs significantly more present in *H. osmophila* and the PUCV-VBL biocontrol were 1-butanol 3-methyl, propanoic acid ethyl ester, ethyl acetate, phenylethyl alcohol, isobutyl acetate, and hexanoic acid ethyl ester (Figure 7). It is almost certain that their antipathogenic effects are due to this overexpression.

In total, the PUCV-VBL biocontrol VOCs were identified as ethyl acetate, propanoic acid ethyl ester, 1-butanol 3-methyl, 1-butanol, 2-methyl, propanoic acid 2-methyl ethyl ester, isobutyl acetate, butanoic acid ethyl ester, 2-butenoic acid ethyl ester (Z), butanoic acid 3-methyl ethyl ester, 1-butanol 3-methyl acetate, 1-butanol 3-methyl acetate, 1-butanol 2-methyl acetate, furan 2-pentyl, hexanoic acid ethyl ester, undecane, phenyl ethyl alcohol, octanoic acid ethyl ester, acetic acid 2-phenylethyl ester, tetradecane, and diethyl phthalate (Supplementary Material, Table S2). These compounds—mostly esters (65%), and to a lesser extent, alcohols (15%), alkanes (15%), and furan (5%)—have all been previously shown to have antifungal activity [28,29,38,39,40,41,42,43,44,45,46,47,48,49,50,51]. The low proportion of furan is concordant with other studies of, e.g., *Streptomyces albulus* NJZJSA2 against *S. sclerotiorum* and *Fusarium oxysporum* [43].

Mainly, Kwasiborski et al., [52] reported that ethyl acetate might be involved in antimicrobial activity against *B. cinerea* [53]. Indeed, 2-phenylethanol—previously observed as the primary volatile produced by other yeasts, such as *Saccharomyces cerevisiae*—has been shown to control pathogen *Sclerotinia sclerotiorum* in vitro and bean seeds, to have a lethal effect against *A. flavus* and to inhibit the production of aflatoxin in sublethal doses. Furthermore, high concentrations of 2-phenylethanol can cause alterations in the biosynthesis of amino acids and proteins in the mitochondria and the nuclei of fungi and bacteria [17,45,54,55].

Additionally, 2-phenylethanol isolated from *K. apiculata* showed inhibitory activity against green and blue mold in citrus fruits caused by *P. digitatum* and *P. italicum* [19]. It was also shown to play a critical role in the antagonistic activity of *A. pullulans* against postharvest fruit pathogens both in vitro and in vivo [56,57].

Next, PUCV-VBL biocontrol VOCs 1-butanol-2-methyl, 1-butanol-3-methyl, 1-propanol-2-methyl, and phenylethyl alcohol have been previously reported in other fungi, such as *M. albus* [58], *Trichoderma atroviride* [59], *P. expansum* [27], *Glomerella cingulata* [60]; and yeasts, such as *S. cerevisiae* [61], *S. pararoseus* [62], *C. intermedia* [38] and *A. pullulans* [51].

Although various combinations of BCAs and or chemicals have been extensively investigated [63], there has to date been no description of bacteria *Gluconobacter cerinus* and yeast *Hanseniaspora osmophila* reported together, or their effects against bunch rot diseases affecting table grapes. Thus the VOC inhibition activity in this study adds to the results from previous research into BCAs.

For example, our study is supported by past results on *Hanseniaspora uvarum*, which was shown to be an effective BCA in, e.g., controlling fungal rot in strawberries by biofumigation using VOCs [64]. It reduces the natural development of decay in grapes and strawberries and maintains quality parameters [28,57,65,66]. It also exerts biocontrol against chili fruit rot [67] and green mold of postharvest oranges [68]; and inhibits the growth of *B. cinerea* with multiple modes of action, including competition for nutrients and space, host defense induction, morphology change and secondary metabolites [57,65,66,69,70]. Indeed, Moreira et al., [71] identified different VOCs produced by *Hanseniaspora* yeasts during red wine vinifications such as 3-methyl-1-butanol, ethyl acetate, phenylethyl alcohol, butanoic acid, and ethyl ester, similar to results from this study.

Furthermore, the findings in this study are further supported by the literature on alternative fungicides. Li et al. [72,73] found that volatile compounds of *Streptomyces globisporus* JK-1 inhibited spore germination and mycelial growth of *B. cinerea* and *P. italicum* in tomato and *Citrus microcarpa*, respectively. Zheng et al., [42] and Chen et al., [74] indicated that VOCs of *Bacillus* spp. were antagonistic to *B. cinerea*, *C. gloeosporioides*, *P. digitatum*, *P. italicum*, and *P. crusto*. Similar inhibitory effect on conidia germination by VOCs produced by two *Aurebasidium pullulans* yeast strains L1 and L8 with 2-methyl-1-butanol, 3-methyl-1-butanol, 2-phenethyl alcohol, and 2-methyl-1-propanol against five postharvest fruit pathogens in vitro and in vivo [51,56,57,75,76,77].

Notably, the antipathogenic action was shown to be highest for the combinatory PUCV-VBL biocontrol. This finding is similar to Li et al., [78], who attempted to reproduce the spectrum of naturally occurring VOCs of *Ceratocystis fimbriata* in proportions calculated using GC-MS analysis. The pure chemicals or several combinations (butyl, ethyl acetate, and ethanol) did not show any inhibitory effect. According to the authors, inhibition likely remains due to synergistic effects among all *C. fimbriata* VOCs, including molecules not detected using current identification methods. Indeed, VOCs have been known to rely on synergistic effects against phytopathogens [58].

In terms of its mode of action, the PUCV-VBL biocontrol VOCs are nearly a mesosystemic, quasi-systemic, or systemic surface mechanism [79]. While they move as a gas in the layer adjacent to the grapes’ surface, they may also enter to stop pathogenic fungi’ growth. Notwithstanding, there may be additional contributions to BCAs activities from, e.g., the production of diffusible compounds and additional mechanisms for the control of pathogenic fungi [80]. In summary, all the results obtained in these trials indicate that antibiosis is a mode of action of this new consortium [81].

## 4. Materials and Methods

### 4.1. Microorganism and Vegetable Materials

PUCV-VBL biocontrol microorganisms were isolated from the surface of table grapes harvested in commercial farms from the Central Valley of Chile. Bacterium *Gluconobacter cerinus* strain 515 (access code RGM2215) and yeast *Hanseniaspora osmophila* strain 337 (access code RGM2214) were obtained and deposited in the Chilean Collection of Microbial Genetic Resources (Patent WO2017088081A1). Microorganisms were identified visually, by morphology, and genetically, by genome amplification with GluF/R primer, for *G. cerinus* [82] and D1/2, for *H. osmophila* [83]. Cell concentration was adjusted to 1 × 10^6^ UFC mL^−1^ for the bacterium using a spectrophotometer at an OD580 nm (S-300, BOECO, Germany) and 1 × 10^4^ UFC mL^−1^ for the yeast using a Neubauer hemocytometer (8100204, Hirschmann, Germany).

Pathogenic fungi under study *Botrytis cinerea*, *Aspergillus tubingensis*, *Penicillium expansum* and *Rhizopus stolonifer* were amplified and sequenced with the following primers: ITS4/5 for the ITS region [84] and βt for ß-tubulin [85]. After identification, samples of each fungal pathogen were coded and stored in ceparium of PUCV Phytopathology Laboratory (Supplementary Material, Table S1).

Each fungal pathogen was adjusted to 1 × 10^5^ conidia mL^−1^. Bunches from table grape cultivars—Thompson Seedless, Crimson Seedless, and Red Globe—were obtained from orchards located in Valparaíso Region (central valley of Chile). Total soluble solids (Brix) defined the harvest date: for cv. Red Globe and Crimson Seedless, 17°Brix; and for cv. Thompson Seedless, 16°Brix. Refrigerated at 1 °C until use, fruit sample surfaces were disinfected by dipping into 1% (*v*/*v*) of sodium hypochlorite (NaOCl) solution for 2 min, rinsed with sterilized water, and then air-dried.

### 4.2. In Vitro Assays of Biological Consortium VOCs

The VOCs of the PUCV-VBL biocontrol and those of its respective components were evaluated against the mycelial growth of summer bunch rot pathogens as follows. The double plate method (Scheme 1a) was used with Mannitol Yeast Peptone (MYP) culture medium (25 g/L mannitol, 5 g/L yeast extract, 3 g/L peptone, and 12 g/L agar) for bacteria; Honey Peptone Agar (HPA), modified (80 g/L honey, 20 g/L peptone, 20 g/L agar) for the yeast [86]; and Potato Dextrose Agar (PDA) (DifcoTM) for pathogens [27,28]. Petri dishes with PDA and MYP culture media were inoculated first with a suspension of each pathogen (20 µL) and then with 100 µL of each biocontroller at the concentrations described above. For each treatment, performed in triplicate and repeated twice, the two plates were sealed with parafilm and incubated for five days at 24 °C [38]. Fungus growth area was measured using image analysis with Image J^®^ program (v. 1.50i, MD, USA). The percentage of inhibition was calculated as ((Control value-Treatment value)/(Control value)) × 100.

### 4.3. In Vivo Assays of Biological Consortium VOCs

Two trials determined in vivo biocontrol VOC production for the PUCV-VBL biocontrol and its components. Following Huang et al. [38], in the first trial (Scheme 1b), two Petri dishes with biocontrollers were placed at the bottom of a humid chamber (21 × 15 × 5 cm), underneath a plastic mesh rack. Six grapes were artificially wounded by a sterile needle, inoculated with pathogenic fungi, and placed on the mesh. In the second trial (Scheme 1c), Petri dishes were replaced with four berries inoculated with the biocontroller. Percent inhibition was calculated as indicated for in vitro assays (though in this case, concerning the berry). Both trials were repeated twice, and each treatment, performed in triplicate.

### 4.4. Identification of Biocontroller-Produced VOCs (SPME-GC-MS)

VOCs produced by biological control agents (BCAs) were identified by Solid-Phase Microextraction followed by Gas Chromatography-Mass Spectrometry. Briefly, samples of grape cultivars (2 g) were taken, deposited in 20 mL vials, and inoculated with 100 µL of each biocontroller at the concentrations described above; pre-incubated for one min at 30 °C with shaking; and then incubated at 24 °C for three days. Before testing, SPME fiber (SU57298U, 50/30 μm DVB/Carboxen/PDMS 23 Ga, Agilent Technologies, Santa Clara, CA, USA) was conditioned at 200 °C for 1 min. Fiber and samples were incubated for 1 h at 30 °C at 40 mm vial depth distance. Retained compounds were desorbed into the chromatograph injection port at 240 °C for 3 min in Splitless mode with a 50 mL min^−1^ purge flow to a Split valve for 0.5 min (Agilent 7890B-5977A single quadrupole MS and PAL3 autosampler) with 5190-4048 liner and 5182-3442 Merlin Microseal. The fiber was reconditioned at 200 °C for 5 min before proceeding with a new adsorption/desorption cycle.

Chromatography was performed following Qin et al. [28]. Helium was used as a carrier gas with a flow of 1 mL min^−1^ and an approximate pressure of 7.7 psi. The column used was a 122-5532 DB-5ms 30 m × 250 µm × 0.25 µm (Agilent Technologies, Santa Clara, CA, USA). The mass spectrometer transfer line was 280 °C, and the quadrupole temperature and the ionization source were set at 150 °C and 230 °C, respectively. The mass spectrometer was operated in SCAN mode. Mass spectra were generated in a range of 50–600 *m*/*z* and at a scan rate of 2.7 cycles per second. Peaks were identified using the NIST14 library in MassHunter Quantitative software.

### 4.5. Statistical Analysis

Data were analyzed by one-way analysis of variance (ANOVA, Prism Software version 6.0) and means compared by Tukey’s test. Statistical significance was assessed at the level of *p* ≤ 0.05. Hierarchical clustering and principal component analyses (PCA) were performed using MetaboAnalyst 5.0 (http://www.metaboanalyst.ca) (accessed on 20 May 2021).

## 5. Conclusions

The results obtained in this work demonstrate the effectiveness of the PUCV-VBL biocontrol VOCs in controlling causal agents of diseases affecting table grapes, including gray and summer bunch rot, under both in vitro and in vivo conditions. The use of these BCAs as a sustainable alternative for the management of phytopathogenic diseases offers numerous advantages, including safe application methods, for the effective control and management of fungal diseases.

## 6. Patents

Patent N° 61580 February 07 2021, WO2017088081A1.

## Figures and Tables

**Table 1:** Mycelial growth inhibition (percentage) determined by in vitro assay by VOCs emitted by *G. cerinus*, *H. osmophila* and PUCV–VBL biological consortium. Means in rows followed by the same letters are not significantly different according to Tukey’s test (*p* = 0.05).

	*G. cerinus*	*H. osmophila*	PUCV-VBL
*B. cinerea*	32 ± 15 ^a^	39 ± 26 ^a^	86 ± 4 ^b^
*A. tubingensis*	36 ± 2 ^a^	53 ± 16 ^a^	52 ± 1 ^a^
*P. expansum*	50 ± 13 ^a^	68 ± 1 ^a^	55 ± 2 ^a^
*R. stolonifer*	18 ± 3 ^a^	29 ± 8 ^a,b^	35 ± 7 ^b^

^a,b^ *p*-value < 0.05, *p*-value was calculated by Tukey’s *t*-test.

**Table 2:** Percentage of inhibition of mycelial growth in vitro of different causal agents with grape-plate test. Means in rows followed by the same letters are not significantly different according to Tukey’s test (*p* = 0.05).

Cultivar	Pathogen	Biocontrol Agents (BCAs)		
		*G. cerinus*	*H. osmophila*	PUCV-VBL
Red Globe	*B. cinerea*	82 ± 9 ^a^	76 ± 14 ^a^	79 ± 15 ^a^
	*A. tubingensis*	75 ± 22 ^a,b^	68 ± 23 ^a^	93 ± 11 ^b^
	*P. expansum*	71 ± 12 ^a^	66 ± 14 ^a^	78 ± 12 ^a^
	*R. stolonifer*	82 ± 12 ^a^	73 ± 10 ^a^	84 ± 15 ^a^
Thompson seedless	*B. cinerea*	54 ± 22 ^a^	75 ± 11 ^b^	79 ± 8 ^b^
	*A. tubingensis*	77 ± 16 ^a^	73 ± 7 ^a^	71 ± 13 ^a^
	*P. expansum*	77 ± 22 ^a^	78 ± 9 ^a^	85 ± 2 ^a^
	*R. stolonifer*	68 ± 24 ^a^	71 ± 22 ^a^	76 ± 17 ^a^
Crimson seedless	*B. cinerea*	82 ± 9 ^b^	54 ± 21 ^a^	86 ± 12 ^b^
	*A. tubingensis*	64 ± 21 ^a,b^	57 ± 18 ^a^	75 ± 14 ^b^
	*P. expansum*	50 ± 11 ^a^	49 ± 15 ^a^	65 ± 13 ^b^
	*R. stolonifer*	89 ± 13 ^b^	60 ± 20 ^a^	64 ± 14 ^a^

^a,b^ *p*-value < 0.05, *p*-value was calculated by Tukey’s *t*-test.

**Table 3:** Percentage of mycelial inhibition of four causal agents in three different table grape cultivars tested in vivo with grapes-on-grapes test. Means in rows followed by the same letters are not significantly different according to Tukey’s test (*p* = 0.05).

Cultivar	Pathogen	*G. cerinus*	*H. osmophila*	PUCV-VBL
Red Globe	*B. cinerea*	85 ± 17 ^a^	83 ± 15 ^a^	83 ± 12 ^a^
	*A. tubingensis*	56 ± 10 ^a^	51 ± 13 ^a^	38 ± 15 ^a^
	*P. expansum*	50 ± 17 ^a^	44 ± 7 ^a^	46 ± 10 ^a^
	*R. stolonifer*	44 ± 15 ^a^	32 ± 20 ^a^	45 ± 19 ^a^
Thompson seedless	*B. cinerea*	96 ± 2 ^a^	96 ± 2 ^a^	98 ± 1 ^a^
	*A. tubingensis*	94 ± 7 ^b^	73 ± 4 ^a^	95 ± 4 ^b^
	*P. expansum*	75 ± 14 ^a^	81± 9 ^a,b^	93 ± 4 ^b^
	*R. stolonifer*	84 ± 14 ^a^	83 ± 15 ^a^	91 ± 3 ^a^
Crimson seedless	*B. cinerea*	51 ± 19 ^a^	34 ± 15 ^a^	72 ± 10 ^b^
	*A. tubingensis*	56 ± 16 ^a^	65 ± 12 ^a^	53 ± 17 ^a^
	*P. expansum*	37 ± 14 ^a^	41 ± 17 ^a^	57 ± 17 ^a^
	*R. stolonifer*	54 ± 17 ^a^	66 ± 4 ^a^	89 ± 20 ^a^

^a,b^ *p*-value < 0.05, *p*-value was calculated by Tukey’s *t*-test.

## Data Availability

The data presented in this study are available in the article.

## Supplementary Materials

The following are available online at https://www.mdpi.com/article/10.3390/antibiotics10060663/s1, Table S1: Molecular identification, percentage identity and access number of GenBank sequences of microorganism used, Table S2: Volatile organic compounds detected from PUCV-VBL consortium on grapes using SPME-GCMS.
