# Supplementary material for: Volatile Organic Compounds (VOCs) Produced by Gluconobacter cerinus and Hanseniaspora osmophila Displaying Control Effect against Table Grape-Rot Pathogens
Source: Antibiotics (Basel). 2021 Jun 1;10(6):663. doi: 10.3390/antibiotics10060663 (PMC8226828; doi:10.3390/antibiotics10060663)
Supplement: Supplementary file 1 [file antibiotics-10-00663-s001.zip › antibiotics-1212304-supplementary.pdf]

antibiotics

MDPI

Table S1. Molecular identification, percentage identity and access number of GenBank sequences of microorganism used. ITS: ITS region primers; BT: beta-tubulin primers.

Code	Organism	Identity % (Match accession)		GenBank ID	
		ITS	BT	ITS	BT
**PUCV1003**	*Botrytis cinerea*	100 (MN522391)	99.74 (MH680911)	MT218334	MT228634
PUCV1024	*Penicillium expansum*	100 (KP204877)	99.31 (KU507296)	MT218335	MT228635
PUCV1074	*Aspergillus tubingensis*	99.49 (MK761046)	99.78 (MK159765)	MT218336	MT228636
PUCV1125	*Rhizopus stolonifer*	99.63 (AY625075)	-	MT227125	

Table S2. Volatile organic compounds detected from PUCV-VBL consortium on grapes using SPME-GCMS.

RT (min)	Main components	Match	R Match	Prob %	RI	Classification	Antifungal activity	Reference
1,79	Acetic acid methyl ester	982	982	70,2	526	ester	No	
1,91	1-Propanol	881	969	70,2	556	alcohol	No	
2,26	Ethyl Acetate	837	853	95,9	612	ester	Yes	[44]; [45]
3,38	Propanoic acid ethyl ester	968	969	97,8	709	ester	Yes	[28]
3,42	n-Propyl acetate	951	953	96,6	708	ester	No	
3,87	1-Butanol 3-methyl	962	962	65,8	736	alcohol	Yes	[44, 46]
3,93	1-Butanol 2-methyl	954	954	41,3	739	alcohol	Yes	[45]
4,31	Propanoic acid 2-methyl ethyl ester	944	944	97,3	755	ester	Yes	[86]; [29]
4,65	Isobutyl acetate	975	975	96,8	771	ester	Yes	[50]
5,32	Butanoic acid, ethyl ester	963	963	96,8	802	ester	Yes	[44]
6,54	2-Butenoic acid ethyl ester (Z)	758	758	59	830	ester	Yes	[41]
6,66	Butanoic acid 2-methyl ethyl ester	959	959	97,2	849	ester	No	
6,81	Butanoic acid 3-methyl ethyl ester	828	828	76,3	854	ester	Yes	[44, 47]
7,49	1-Butanol 3-methyl acetate	970	970	92,1	876	ester	Yes	[48]
7,54	1-Butanol 2-methyl acetate	942	943	94,6	880	ester	Yes	[86]

11,06	Furan 2-pentyl	899	899	85,5	993	furan	Yes	[49]
11,36	Hexanoic acid ethyl ester	944	944	92,2	1000	ester	Yes	[44]
14,55	Undecane	769	931	27,7	1100	alkane	Yes	[51]
14,91	Phenylethyl Alcohol	956	962	85,7	1116	alcohol	Yes	[42]
17,45	Octanoic acid ethyl ester	890	890	95,9	1196	ester	Yes	[38]
17,56	Dodecane	973	974	65,6	1200	alkane	No	
17,93	Undecane 2,6-dimethyl	938	939	50,3	1210	alkane	No	
19,12	Acetic acid 2-phenylethyl ester	963	963	50,6	1258	ester	Yes	[44]
23,08	Tetradecane	962	968	56,1	1400	alkane	Yes	[39]
27,67	Diethyl Phthalate	918	918	52,4	1594	ester	Yes	[40]
27,84	Dodecanoic acid, ethyl ester	724	739	92,8	1595	ester	No	
